# Chemical Bonding Topology of Metal-Centered Polygonal Wheels: Two-Dimensional Analogues of Metallaboranes Related to Benzene and Cyclopentadienide

**DOI:** 10.1021/acs.inorgchem.3c00267

**Published:** 2023-03-23

**Authors:** R. Bruce King

**Affiliations:** Department of Chemistry, University of Georgia, Athens, Georgia 30602, United States

## Abstract

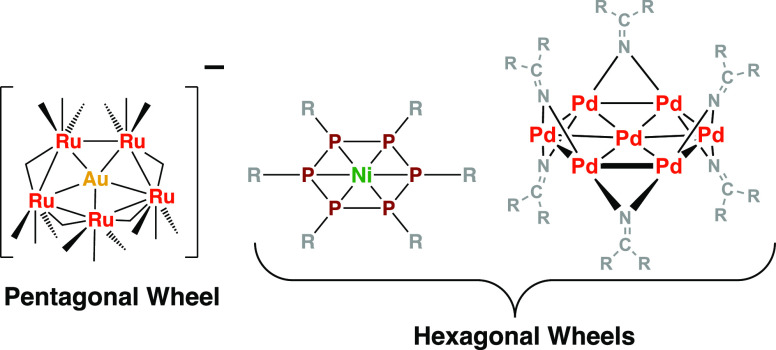

The anion [Au@Ru_5_(CO)_15_(μ-CO)_4_]^−^ has a pentagonal wheel structure that can be derived from a hypothetical pentagonal ruthenium carbonyl cluster Ru_5_(CO)_20_ by insertion of a gold atom in the center, thereby splitting the original Ru_5_ pentagon in Ru_5_(CO)_20_ into five AuRu_2_ triangles. The six electrons used to form 3c–2e bonds in three of the five AuRu_2_ triangles suggest a relationship to the aromatic sextet of the likewise pentagonal cyclopentadienide anion. Furthermore, the pentagonal wheel framework of [Au@Ru_5_(CO)_15_(μ-CO)_4_]^−^ can be derived from a pentagonal bipyramid, such as that found in the deltahedral borane anion B_7_H_7_^2–^, by bringing the two C_5_ axial vertices together at the center of the equatorial pentagon. Similarly, the hexagonal wheel complexes Ni@P_6_R_6_ and Pd@Pd_6_(μ-N=CtBu_2_)_6_ with six triangular faces can be derived from a hexagonal bipyramid, such as that found in the dirhenaborane (η^5^-Me_5_C_5_)_2_Re_2_B_6_H_4_Cl_2_, by bringing the two C_6_ axial vertices together at the center of the equatorial hexagon. A reasonable chemical bonding model for the hexagonal wheel complexes has three-fold symmetry with 3c–2e bonds in three of these six triangular faces analogous to the C=C double bonds in a Kekulé structure of benzene.

## Abstract 2

Chemical bonding models are presented for the pentagonal wheel anion [Au@Ru_5_(CO)_15_(μ-CO)_4_]^−^ and the neutral hexagonal wheel structures Ni@P_6_R_6_ and Pd@Pd_6_(μ-N=CtBu_2_)_6_ that indicate them to be analogues of the cyclopentadienide anion and benzene, respectively. These polygonal wheel molecules are also shown to be two-dimensional analogues of three-dimensional polyhedral boranes.

## 1 Introduction

**Figure 1:**
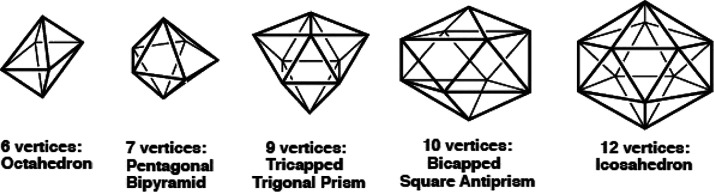
Some examples of deltahedra found in polyhedral borane structures.

An important class of boron compounds consists of the polyhedral boranes and their derivatives including metallaboranes. Such species exhibit three-dimensional structures based on polyhedra having exclusively or mainly triangular faces.^1,2^ Such polyhedra having exclusively triangular faces are conveniently known as deltahedra (Figure 1). Aspects of the chemical bonding topology in these deltahedra classify them as three-dimensional aromatic systems.^3,4^ This aromaticity is reflected in their unusual stability relative to other borane derivatives. This is particularly true of icosahedral derivatives such as B_12_H_12_^2–^ and C_2_B_10_H_12_.

In a crude sense, the three-dimensional aromaticity in such deltahedral boranes makes them analogues of the iconic two-dimensional aromatic compound benzene as well as related species such as cyclopentadienide, C_5_H_5_^–^, and tropylium, C_7_H_7_^+^. However, unlike these two-dimensional carbocyclic aromatic species, the three-dimensional aromatic deltahedral boranes have boron triangles as fundamental building blocks. Two-dimensional aromatic systems having triangles as building blocks are much less common and have a much more recent history. The simplest such species are polygonal wheels in which a central atom (the hub) is surrounded by a polygon of atoms (the periphery or rim). In order to maintain a two-dimensional structure, a planar hybridization scheme is required for the central atom. Such *n*-gonal wheels can be regarded as planar structures with *n* + 1 vertices and *n* triangular faces. The group 10 transition metals nickel, palladium, and platinum as well as the coinage metals are ideally suited for the central atom since they can exhibit two-dimensional coordination geometries through trigonal planar or square planar hybridization.

**Figure 2:**
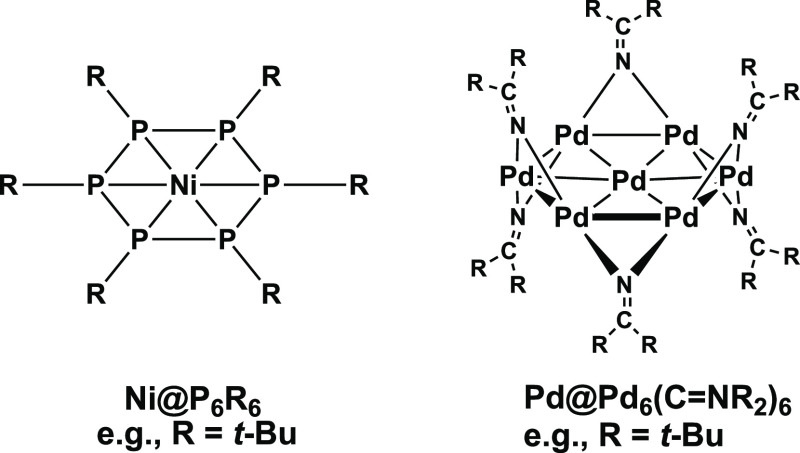
Some experimentally known hexagonal wheel structures.

The first example of stable polygonal wheel species was the hexagonal wheel Ni@P_6_Bu^t^_6_, originally reported by Ahlrichs et al. in 1992^5^ and shown by X-ray crystallography to have a hexagonal wheel structure with peripheral phosphorus atoms and a central nickel atom (Figure 2). Much more recently, in 2020, Hayton and co-workers discovered a second hexagonal wheel species, namely, the palladium cluster Pd@Pd_6_(μ-N=CBu^t^_2_)_6_ separated from the reaction of (CH_3_CN)_2_PdCl_2_ with Li[N=CBu^t^_2_] (Figure 2).^6^ The hexagonal wheel structure of the Pd@Pd_6_(μ-N=CBu^t^_2_)_6_ molecule is particularly interesting since both the rim and the center of the wheel are the same atom, namely, palladium. Other examples of hexagonal wheel structures include the xenophilic manganese carbonyl anion [Mn@Mn_6_(thf)_6_(CO)_12_]^−^ containing a central Mn@Mn_6_ wheel,^7,8^ the cobalt cluster Co@Co_6_(μ_3_-H)_6_{μ-N(SiMe_3_)_2_}_6_ with hydrogen atoms capping each of the triangular faces of the Co@Co_6_ hexagonal wheel,^9^ and Pd[Re_2_(CO)_8_(μ-SbPh_2_)(μ-H)_2_]_2_ containing a Pd@Re_4_Sb_2_ hexagonal wheel with edge bridging hydrogen atoms. In addition, the bimetallic copper iron carbonyl cluster trianion [Cu_5_Fe_4_(CO)_16_]^3–^ contains a central Cu@Cu_4_Fe_2_ hexagonal wheel with Fe(CO)_4_ units bridging two opposite Cu–Cu edges.^10^

**Figure 3:**
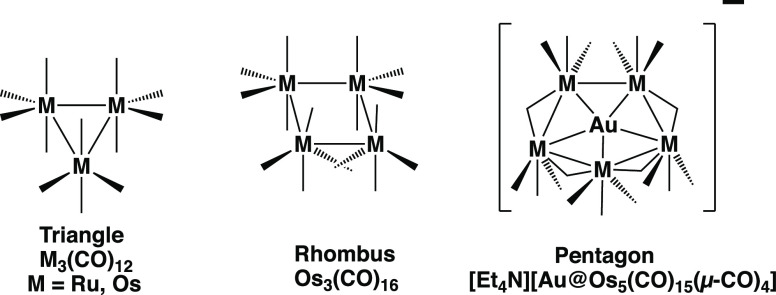
Progression from triangular M_3_(CO)_12_(M = Ru, Os) to rhombus Os_4_(CO)_16_ and pentagonal [Et_4_N][Au@Ru_5_(CO)_15_(μ-CO)_4_] involving experimentally known and structurally characterized species.

A key development in the further evolution of the chemistry of metal-centered wheels is the very recent discovery of the gold-centered polygonal ruthenium carbonyl wheel in the anion [Au@Ru_5_(CO)_15_(μ-CO)_4_]^−^, structurally characterized as its tetraethylammonium salt.^11^ This pentagonal wheel species is particularly significant in representing the third member of an experimentally realized series of polygonal polynuclear carbonyl derivatives of the second and third row group 8 transition metals starting with the triangular M_3_(CO)_12_ (M = Ru,^12^ Os^13,14^) followed by the rhombus^15,16^ Os_4_(CO)_16_ that have been synthesized and structurally characterized by X-ray crystallography (Figure 3). Assuming the ruthenium atoms in [Au@Ru_5_(CO)_15_(μ-CO)_4_]^−^ to be zerovalent as they are in Ru_3_(CO)_12_ and Ru_4_(CO)_16_ leads to a −1 formal oxidation state for the central gold atom similar to that in the experimentally known cesium auride, CsAu.^17,18^ Thus, the anion [Au@Ru(CO)_15_(μ-CO)_4_]^−^ can alternatively be viewed as a complex of the auride anion with pentagonal planar coordination to five ruthenium carbonyl “ligands”.

## 2 Results and Discussion

### 2.1 Chemical Bonding in the Pentagonal Metal Carbonyl Wheel Anion [Au@Ru_5_(CO)_15_(μ-CO)_4_]^−^

The group 10 metal atoms in polygonal metal carbonyl derivatives of the stoichiometry [M(CO)_4_]_*n*_ (M = Ru, Os; *n* = 3, 4) attain the favored 18-electron configuration as follows:central metal atom (Ru or Os)8 electrons4 CO groups: 4 × 2 =8 electronsM–M bonds to the two neighboring
metals2 electronstotal metal electrons18 electrons

**Figure 4:**
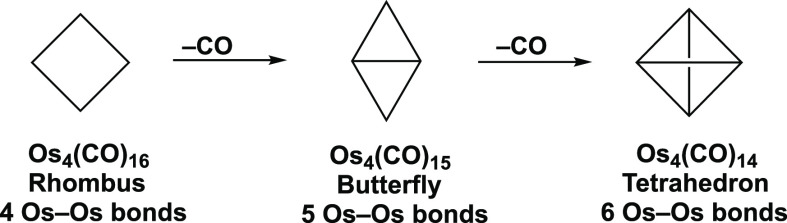
Addition of Os–Os bonds upon carbonyl loss in the series Os_4_(CO)_16_ → Os_4_(CO)_15_ → Os_4_(CO)_14_.

The triangular species M_3_(CO)_12_ (M = Ru, Os) are stabilized further by σ-aromaticity^19^ similar to that in cyclopropane.^20−25^ However, the M_4_ rhombus in M_4_(CO)_16_ is antiaromatic like the C_4_ ring in cyclobutane. This is reflected experimentally in the instability of rhombus Os_4_(CO)_16_, which decomposes in solution under an inert atmosphere at room temperature in a complicated reaction leading to the very stable Os_3_(CO)_12_.^13,14^ However, loss of CO groups from Os_4_(CO)_16_ to give first Os_4_(CO)_15_ with a butterfly structure consisting of two triangles sharing an edge and then Os_4_(CO)_14_ with a central Os_4_ tetrahedron leads to much more stable structures (Figure 4). The successive losses of carbonyl groups from Os_4_(CO)_16_ result in the formation of new Os–Os bonds leading to the Os_4_ rhombus in Os_4_(CO)_16_, the Os_4_ butterfly in Os_4_(CO)_15_, and finally the Os_4_ tetrahedron in Os_4_(CO)_14_ having four, five, and six Os–Os bonds, respectively. These experimental observations demonstrate the value of σ-aromatic metal triangles in stabilizing metal carbonyl cluster structures.

**Figure 5:**
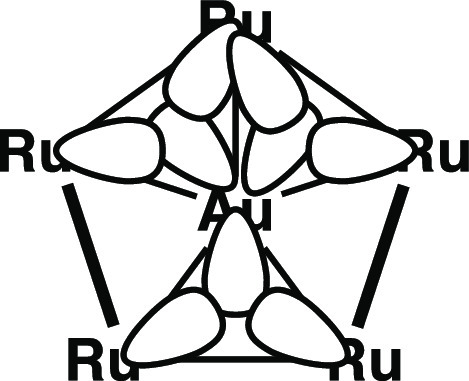
Possible combination of 3c–2e Ru_2_Au bonds and 2c–2e Ru–Ru bonds in the Au@Ru_5_ pentagonal wheel of [Au@Ru_5_(CO)_15_(μ-CO)_4_]^−^.

**Figure 6:**
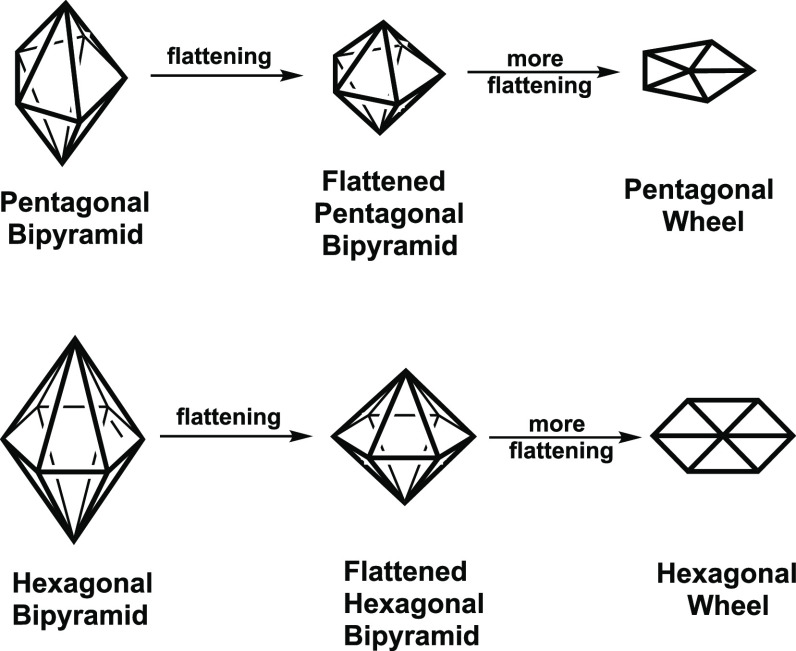
Flattening of polygonal bipyramids to polygonal wheels.

Adding a central metal atom to a larger outer polygon to generate a wheel structure splits the polygon into a set of triangles thereby stabilizing the system through σ-aromaticity of the metal triangles. The recently synthesized anion [Au@Ru_5_(CO)_15_(μ-CO)_4_]^−^ as its stable tetraethylammonium salt is an excellent example.^11^ The Ru_5_ pentagon in a hypothetical Ru_5_(CO)_20_ is dissected into five Ru_2_Au triangles by interaction with the central gold atom. In the [Au@Ru_5_(CO)_15_(μ-CO)_4_]^−^ structure, all five ruthenium atoms effectively retain the favored 18-electron configuration of the hypothetical Ru_5_(CO)_20_ with the two electrons of the “missing” carbonyl group being replaced by one electron from the central gold atom and one electron coming from the negative charge on the anion. The central gold atom can formally be considered to have trigonal planar hybridization with its three gold orbitals converting three of the five Ru–Ru two-center two-electron (2c–2e) bonds in the Ru_5_ pentagon into three-center two-electron (3c–2e) Ru_2_Au bonds in a localized bonding model (Figure 5). Note that the two-dimensional pentagonal wheel found in [Au@Ru_5_(CO)_15_(μ-CO)_4_]^−^ can be generated by flattening the three-dimensional pentagonal bipyramid found in borane species such as the B_7_H_7_^2–^ dianion. This flattening process brings the two axial vertices together as the central vertex of the pentagonal wheel (Figure 6).

### 2.2 From Tris(Olefin) Metal Complexes to Hexagonal Wheel Structures

**Figure 7:**
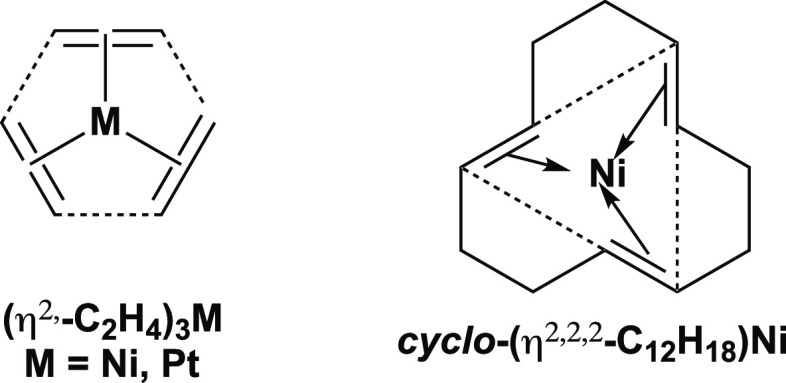
Tris(olefin) complexes of the group 10 metals. The dashed lines are the three non-bonding long edges in the C_6_ hexagons surrounding the central metal atom.

A sequence of triangular, square, and pentagonal precursor structures similar to that for the ruthenium carbonyl clusters (Figure 3) is not known for the two experimentally realized hexagonal wheel structures, namely, Ni@P_6_But_6_ and Pd@Pd_6_(μ-N=CBu^t^_2_)_6_. Instead, the hexagonal nickel wheel Ni@P_6_But_6_ can be related to the experimentally known tris(ethylene) complexes (η^2^-C_2_H_4_)_3_M (M = Ni,^26^ Pt^27−29^ as well as the 1,5,9-cyclododecatriene complex (η^2,2,2^-C_12_H_18_)Ni obtained by the trimerization of butadiene^30^ in which a trigonal planar sp^2^-hybridized group 10 metal is coordinated to three C=C double bonds (Figure 7). Such nickel(0) trisolefin complexes can also be interpreted as M@C_6_ structures in which the group 10 metal is located in the center of a distorted carbon hexagon with alternating short (bonding) and long (nonbonding) edges. Thus the short edges in such hexagons correspond to the coordinated C=C double bonds, whereas the long edges are the nonbonding distances between a given olefinic ligand carbon and the nearest carbon atom in an adjacent olefinic ligand (dashed lines in Figure 7). An undistorted C_6_ hexagon, such as that found in benzene, is too small to encapsulate a group 10 metal atom to form a hexagonal wheel structure with outer carbon atoms and the metal atom in the center. Thus, an analogous nickel–carbon hexagonal wheel of the type Ni@C_6_H_12_ is not viable since if the Ni–C bonds have a favorable length of 1.85 Å, geometry requires that the C–C bond lengths also be 1.85 Å, which is much too long for effective bonding.

Replacing the carbon vertices of these tris(olefin) structures of the group 10 metals with larger atoms can produce hexagons large enough to encapsulate the central metal atom to form wheel-like structures. This was first realized in the synthesis of the hexagonal wheel structure Ni@P_6_Bu^t^_6_ (Figure 2).^5^ Thus, increasing the size of the peripheral atoms from carbon to phosphorus is enough to provide a peripheral hexagon large enough to enclose a central nickel atom. The chemical bonding in such molecular wheels was examined by molecular orbital methods shortly after the discovery of Ni@P_6_Bu^t^_6_ as well as more recently by topology-based methods providing a valence-bond description.^31^

### 2.3 Chemical Bonding in Hexagonal Wheel Complexes

**Figure 8:**
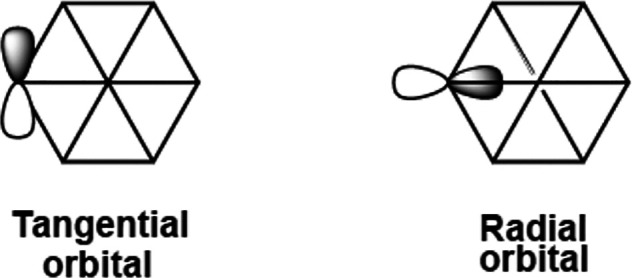
Internal orbitals of the peripheral atoms in hexagonal wheel structures.

**Figure 9:**
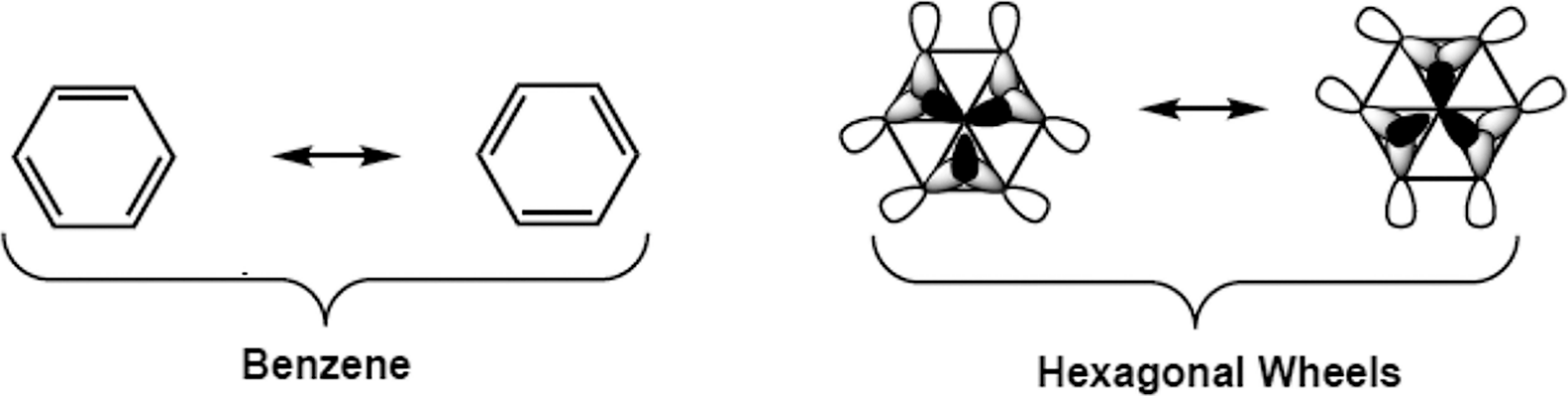
Comparison of the canonical structures of benzene and of the hexagonal wheels.

Consider first the hexagonal phosphorus wheel Ni@P_6_R_6_ in which each phosphorus vertex is considered as contributing two internal orbitals, namely, a tangential p orbital and a radial orbital (Figure 8). The radial orbital represents one part of trigonal sp^2^ hybridization of the peripheral phosphorus atom, which also includes a P–R bond and a stereochemically active lone pair. The tangential p orbitals on the six phosphorus atoms overlap to form three bonding orbitals, each containing an electron pair, as well as three empty antibonding orbitals. The central nickel atom in Ni@P_6_R_6_ exhibits trigonal planar sp^2^ hybridization. Each of the three sp^2^ hybrid orbitals from the central nickel atom overlaps with the radial orbitals of two adjacent peripheral phosphorus atoms to form a three-center two-electron NiP_2_ bond. Thus, the skeletal bonding topology of the Ni@P_6_R_6_ phosphorus hexagonal wheels consists of an arrangement of 3c–2e bonds in nonadjacent triangles preserving three-fold symmetry (Figure 9). The two possible ways of filling three nonadjacent NiP_2_ triangles represent canonical structures that are the analogues of the two canonical cyclohexatriene Kekulé structures in benzene. The six electrons involved in the three 3c–2e NiP_2_ bonds in a localized chemical bonding topology of Ni@P_6_R_6_ can be considered as corresponding to the aromatic sextet in benzene.

Now consider the more complicated hexagonal palladium wheel Pd@Pd_6_(μ-N=CBu^t^_2_)_6_. The peripheral palladium atoms exhibit linear N–Pd–N coordination to two ketenimine nitrogen atoms requiring two sp hybrid orbitals. The N–Pd–N chains are not perpendicular to the plane of the Pd_6_ hexagon so that each coordinating ketenimine carbon atom can bridge two adjacent palladium atoms. The two p orbitals remaining from the sp^3^ manifolds of each peripheral palladium atom after forming the linear sp hybrids for the N–Pd–N chain are the internal orbitals for the skeletal bonding within the Pd@Pd_6_ hexagonal wheel. Similar to Ni@P_6_R_6_ discussed above, one of these p orbitals on each palladium atom is a tangential orbital oriented along the hexagonal periphery to form three bonding and three antibonding orbitals. The other p orbital on each peripheral palladium atom is a radial orbital oriented toward the central palladium atom. Overlap of two radial orbitals from adjacent peripheral palladium atoms with an sp^2^ hybrid from the central palladium atom forms a 3c–2e bond in one of the six Pd_3_ triangles of the Pd@Pd_6_(μ-N=CBu^t^_2_)_6_ structure. In this way, the skeletal bonding topology of 3c–2e bonding in three nonadjacent Pd_3_ triangles in Pd@Pd_6_(μ-N=CBu^t^_2_)_6_ preserving three-fold symmetry is completely analogous to the skeletal bonding in the Ni@P_6_R_6_ phosphorus wheels discussed above. Theoretical studies^6^ on Pd@Pd_6_(μ-N=CBu^t^_2_)_6_ lead to diatropic (negative) nucleus independent chemical shift (NICS) values confirming the aromaticity in these palladium hexagonal wheels.

### 2.4 Polygonal Wheels as Two-Dimensional Analogues of Deltahedral Borane Structures

**Figure 10:**
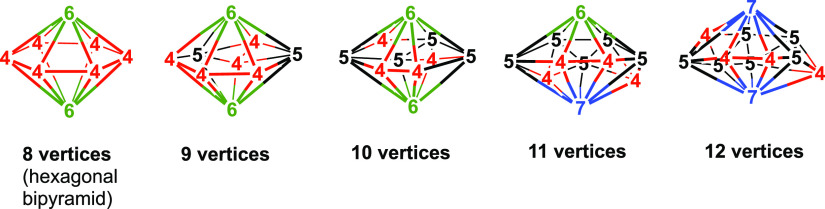
Flattened oblate ellipsoidal central Re_2_B_*n*–2_ deltahedra found in the dirhenaboranes (η^5^-Me_5_C_5_)_2_Re_2_B_*n*–2_H_*n*–2_ (*n* = 8–12). Vertices of degrees 4, 5, 6, and 7 are colored red, black, green, and blue, respectively.

The chemical bonding topology of 3c–2e bonds in half of the triangular faces in a localized bonding model for the Ni@P_6_R_6_ and Pd@Pd_6_(μ-N=CBu^t^_2_)_6_ hexagonal wheels (Figure 9) corresponding to the aromatic sextet of benzene resembles the chemical bonding topology in some types of metallaboranes. This suggests that the hexagonal wheels are two-dimensional analogues of such systems. The chemical bonding topology of metal-free boranes and carboranes of the types B_*n*_H_*n*_^2–^, C_2_B_*n*–1_H_*n*_^–^, and C_2_B_*n*–2_H_*n*_ (*n* = 6–14) consists of the most spherical *closo* deltahedral canonical structures with an *n*-center core bond formed by the vertex radial orbitals and a chain of 2c–2e bonds formed by the vertex tangential orbitals.^4,32^ However, as boron or carbon vertices are replaced by transition metal vertices with suitable external ligands, the underlying deltahedra become less spherical owing to different vertex degree preferences for transition metals and boron or carbon atoms. Thus, the experimentally known so-called *oblatocloso* dirhenaboranes (η^5^-Me_5_C_5_)_2_Re_2_B_*n*–2_H_*n*–2_ (*n* = 8–12)^33−35^ have flattened oblate ellipsoidal structures with low surface curvature at degree 6 and/or 7 rhenium vertices and high surface curvature at degree 4 and/or 5 boron vertices (Figure 10). The chemical bonding topology of such *n*-vertex dirhenaboranes consists of 3c–2e bonds in *n* of the 2*n* – 4 faces of the deltahedron with only a formal Re=Re double bond rather than a multicenter bond in the center of the deltahedron.^36^ Note also that flattening completely the three-dimensional hexagonal bipyramidal structure, known experimentally^35^ in (η^5^-Me_5_C_5_)_2_Re_2_B_6_H_4_Cl_2_, by bringing the two C_6_ axial vertices together at the center of the equatorial hexagon leads to the hexagonal wheel structure (Figure 6). This flattening process converts a hexagonal bipyramidal dirhenaborane structure into a hexagonal wheel structure and indicates a connection between hexagonal wheels and metallaboranes.

The chemical bonding topology in the gold-centered pentagonal wheel anion [Au@Ru_5_(CO)_15_(μ-CO)_4_]^−^ resembles that in the hexagonal wheel complexes Ni@P_6_R_6_ and Pd@Pd_6_(μ-N=CBu^t^_2_)_6_ in having three 3c–2e bonds within the pentagonal wheel (Figure 5). The six electrons in these three 3c–2e bonds of the pentagonal wheel can be related to the aromatic sextet of the cyclopentadienide anion with a similar pentagonal structure.

## 3 Conclusions

The anion [Au@Ru_5_(CO)_15_(μ-CO)_4_]^−^ has a pentagonal wheel structure that can be derived from a hypothetical pentagonal ruthenium carbonyl cluster Ru_5_(CO)_20_ by insertion of a gold atom in the center. This splits the original Ru_5_ pentagon in Ru_5_(CO)_20_ into five AuRu_2_ triangles leading to considerable stabilization through the σ-aromaticity characteristic of three-membered rings. The six electrons used to form 3c–2e bonds in three of the five AuRu_2_ triangles suggest a relationship to the aromatic sextet of the likewise pentagonal cyclopentadienide anion. The pentagonal Ru_5_(CO)_20_ origin of the [Au@Ru_5_(CO)_15_(μ-CO)_4_]^−^ anion can be considered as the third member of a homologous series starting with the experimentally known very stable Ru_3_(CO)_12_ triangle and the relatively unstable Os_4_(CO)_16_ rhombus. Furthermore, the pentagonal wheel framework of [Au@Ru_5_(CO)_15_(μ-CO)_4_]^−^ can be derived from a pentagonal bipyramid, such as that found in the deltahedral borane anion B_7_H_7_^2–^, by bringing the two C_5_ axial vertices together at the center of the equatorial pentagon.

The hexagonal wheel complexes Ni@P_6_R_6_ and Pd@Pd_6_(μ-N=CBu^t^_2_)_6_ with six triangular faces can be derived from a hexagonal bipyramid, such as that found in the dirhenaborane (η^5^-Me_5_C_5_)_2_Re_2_B_6_H_4_Cl_2_, by bringing the two C_6_ axial vertices together at the center of the equatorial hexagon. A reasonable chemical bonding model for the hexagonal wheel complexes has three-fold symmetry with 3c–2e bonds in three of these six triangular faces analogous to the C=C double bonds in a Kekulé structure of benzene.
